# Comparison of different ratios of cefoperazone/sulbactam in patients with pyelonephritis

**DOI:** 10.12669/pjms.39.1.6850

**Published:** 2023

**Authors:** Weizhong Jiang, Lili Cai

**Affiliations:** 1 Weizhong Jiang, Department of Nephrology, Huzhou Central Hospital, Affiliated Central Hospital Huzhou University, Huzhou, Zhejiang Province, 313000 P.R. China; 2 Lili Cai, Department of Nephrology, Huzhou Traditional Chinese Medicine Hospital, Affiliated to Zhejiang Chinese Medical University, Huzhou, Zhejiang Province, 313000 P.R. China

**Keywords:** Cefoperazone/sulbactam, Pyelonephritis, Anti-inflammatory, Bacterial clearance

## Abstract

**Objective:**

To compare the clinical efficacy of different ratios of cefoperazone/sulbactam in the treatment of patients with pyelonephritis.

**Methods:**

In this retrospective study clinical records of patients with pyelonephritis treated in Huzhou Traditional Chinese Medicine Hospital from July 2020 to July 2021 were collected,. It included 55 patients who received cefoperazone/sulbactam 2:1 treatment (Control group) and 57 patients who received 1 cefoperazone/sulbactam 1:1 treatment (Observation group). Clinical response, inflammatory reaction and bacterial clearance were compared between the two groups.

**Results:**

The levels of C-reactive protein (CRP), interleukin-6 (IL-6), interleukin-8 (IL-8) and leukocyte count (WBC) in the observation group were lower than those in the control group (*P*<0.05). The total efficacy of the Observation-group was 92.98%, higher than 80.00% of the control group (*P*<0.05). Fifty-eight strains of bacteria were isolated from the Control-group and 59 strains from the Observation-group. The bacterial clearance rates were 65.52% (38/58) and 83.05% (49/59), respectively. The differences were statistically significant (*P*<0.05).

**Conclusions:**

The clinical efficacy of 1:1 ratio of cefoperazone/sulbactam in the treatment of patients with pyelonephritis was superior that of 2:1 ratio. This ratio allows to fully utilize the antibacterial effect of cefoperazone, with a significant decrease in inflammation markers and an improvement in bacterial clearance.

## INTRODUCTION

Pyelonephritis is a common urinary tract infection caused by various pathogenic microorganisms such as bacteria, viruses, and fungi in the renal pelvis. The pathological manifestations of pyelonephritis include inflammation of the renal interstitium, renal tubules and renal parenchyma, which can cause symptoms such as high fever, chills, frequent urination and dysuria, accompanied by pain on percussion and tenderness over costovertebral angle (renal angle), which largely affects the quality of life of patients.^1^

Cefoperazone is the third generation of cephalosporin antibiotics with a wide antibacterial spectrum and strong effect. However, cefoperazone is high susceptibile to the hydrolysis by plasmid- and chromosome-mediated β-lactamases, resulting in the decline in the curative effect.^2^,^3^ Sulbactam is a β-lactamase inhibitor, which has an irreversible inhibitory effect on β-lactam antibiotic resistant strains. Previous studies have demonstrated the efficacy of cefoperazone/sulbactam combination in treating Gram-negative bacterial infections.^4^,^5^ This combination can effectively inhibit β-lactamase of pathogenic bacteria, enhance the resistance of cefoperazone sodium to β-lactamase hydrolysis, and has an obvious synergistic effect.^4^,^5^ Many studies have also compared the efficacy of combination of cefoperazone and sulbactam with other antibiotics.^6^,^7^ However, there are few studies on the clinical efficacy of different ratios of cefoperazone/sulbactam.

Appropriate antibiotic dosing is crucial for favorable clinical outcomes of bacterial infections. While the dose of sulbactam usually needs to be adjusted according to the renal function of patients, cefoperazone does not require such adjustment. Therefore, the dosage of cefoperazone in the cefoperazone/sulbactam combined treatment regimen may potentially be insufficient.^8^ A study by Chao et al^8^ showed that a low 1:1 cefoperazone/sulbactam ratio was effective in patients with chronic kidney disease. To our knowledge, only Chang et al^9^ has conducted studies comparing the efficiency of different cefoperazone/sulbactam ratios against multidrug resistant organisms. Therefore, the purpose of this retrospective analysis was to compare the clinical efficacy of different ratios of cefoperazone/sulbactam in the treatment of patients with pyelonephritis in order to validate the findings of the previous study and provide more evidence for future research.

## METHODS

This retrospective study analyzed clinical records of 112 patients with pyelonephritis treated in Huzhou Traditional Chinese Medicine Hospital from July 2020 to July 2021. It included 55 patients treated with cefoperazone/sulbactam ratio of 2:1 (the control group) and 57 patients treated with cefoperazone/sulbactam ratio of 1:1 (the observation group). The study was approved by the ethics committee of Huzhou Traditional Chinese Medicine Hospital (Approval no.: 20220808; Date: August 8, 2022), and informed consent was received from all patients.

Patients in the control group received an intravenous injection of 2g of cefoperazone and 1g sulbactam, mixed with 150ml of 0.9% sodium chloride solution, twice a day, for two weeks.

Patients in the observation group received an intravenous injection of 3g cefoperazone/sulbactam (the trade name is “Supushin”, specification: 3.0g, cefoperazone: sulbactam ratio at 1:1), mixed with 150ml 0.9% sodium chloride solution, twice a day for two weeks.

Only patients fulfilling all of the following diagnostic criteria of pyelonephritis^10^ were included: 1) At least one of the symptoms of fever, abnormal urination, dysuria, urgency, frequency, flank pain or costovertebral angle tenderness; 2) Routine urine examination showing white blood cells, red blood cells and urinary protein; 3) The urinary bacterial count is greater than 10^5^/ml.

### Inclusion criteria:

- Complete clinical data
- Patients diagnosed as pyelonephritis according to the diagnostic criteria^10^ listed above
- Did not receive relevant western and traditional Chinese medicine treatment
- Patients who were naïve to antibiotics
- Patients without complicated pyelonephritis^11^
- Age >18 years old

### Exclusion criteria:

- Patients with urethritis, cystitis, urethral syndrome, renal calculi or renal tumor
- Severe underlying diseases like diabetes and hypertension, organ dysfunction and malignant tumors
- Abnormal structure of urinary tract
- Women who were pregnant, lactating or received reproductive treatments in recent three months
- Patients with contraindications to cefoperazone and sulbactam

Patient characteristics and laboratory examination records on the date of admission and the date of completion of the course of treatment were collected. C-reactive protein (CRP), interleukin-6 (IL-6), interleukin-8 (IL-8) and leukocyte count (WBC) were selected as the efficacy evaluation indicators.^12^,^13^ CRP, IL-6 and IL-8 levels were detected by the enzyme-linked immunosorbent assay (reagents were provided by Shanghai enzyme-linked biology). WBC detection was done by visual counting using a microscope. The overall efficacy evaluation was divided into the following four levels:

### Cure:

The symptoms and signs of the patient disappeared completely; routine urine test was performed every two days and the results were negative for three consecutive times ; negative urine culture .

### Remarkable effect:

No symptoms and signs of pyelonephritis detected except one; greater than 50% reduction in WBC per high-power field in the routine urine test; negative urine culture or the colony counts < 10^4^/ml.

### Effective:

At least one of the symptoms and signs was improved; greater than 30% reduction in WBC per high-power field in the routine urine test; positive urine culture.

### Ineffective:

No improvement or aggravation of symptoms and signs, no improvement in the results of routine urine test; positive urine culture.

Total effective rate = (cure+markedly effective+effective)/total.

### Bacterial clearance effect:

Bacterial cultures were obtained from the mid-stream urine samples after the treatment. After culturing, Gram staining was performed, and the pathogenic bacteria were detected by microbial identification and drug sensitivity analysis method. Urine test was done once a week. If the bacteriological culture was negative for two consecutive times, the infection was considered as cleared. If the bacteriological culture after the treatment was similar to that before the treatment, it was considered as not cleared. Detection of one of the two or more kinds of originally cultured pathogenic bacteria was considered as partial clearance (Clearance rate=number of complete clearance/total number of strains×100%).^14^

### Statistical analysis:

SPSS 22.0 was used for data processing and [n (%)] was used to represent non-grade count data. The test method was *χ^2^*, (*χ̄*±*S*) was used to represent measurement data, and a t-test was performed. When *P*<0.05, the difference was considered statistically significant.

## RESULTS

A total of 112 patients were included. Of them, 55 patients (27 males and 28 females; mean age 50.49±13.42 years) were in the control group and 57 patients (22 males and 35 females; mean age 51.94±14.92 years) comprised the observation group. There were no differences in patient characteristics between the two groups (*P*>0.05) (Table-I).

**Table-I:** Comparison of general information between the two groups.

Group	n	Gender (Male/Female)	Age (Year)	Course of disease (months)
Control-group	55	27/28	50.49±13.42	2.47±0.72
Observation-group	57	22/35	51.94±14.92	2.64±0.65
*χ^2^*/*t*	-	1.253	0.542	1.302
*P*	-	0.263	0.589	0.196

Before the treatment, there were no significant differences in the levels of CRP, IL-6, IL-8 and WBC between the two groups (*P*>0.05). After the treatment, the levels of CRP, IL-6, IL-8 and WBC in the two groups were lower than those before the treatment, and the inter-group comparison showed that the levels of CRP, IL-6, IL-8 and WBC in the observation group were lower (*P*<0.05) (Table-II).

**Table-II:** Comparison of laboratory test results before and after treatment between the two groups (*χ̅*±*S*).

Group (n)	CRP (mg/L)		IL-6 (pg/ml)		IL-8 (pg/ml)		WBC (×10^9^/L)	
			
	before therapy	After treatment	before therapy	After treatment	before therapy	After treatment	before therapy	After treatment
Control-group (n=55)	10.17±2.09	5.54±1.41^a^	165.49±12.20	92.47±11.26^a^	265.60±13.84	192.81±12.57^a^	13.12±2.89	6.21±2.01^a^
Observation-group (n=57)	10.78±1.92	3.29±1.48^a^	163.44±12.81	64.15±11.00^a^	269.22±17.42	158.14±15.86^a^	13.96±2.86	3.70±1.54^a^
t	1.602	8.229	0.867	13.459	1.218	12.790	0.846	7.429
P	0.112	<0.001	0.388	<0.001	0.226	<0.001	0.126	<0.001

***Note:*** Compared with this group before treatment,

a *P*<0.05.

Fifty-eight strains of bacteria were isolated from the control group and 59 strains of bacteria were isolated from the observation group, with bacterial clearance rates of 65.52% (38/58) and 83.05% (49/59), respectively. The difference was statistically significant (*P*<0.05) (Table-III). In terms of overall efficacy, the total effective rate of the observation-group was 92.98%, which was higher than the 80.00% of the control group (*P*<0.05) (Table IV).

**Table-III:** Bacterial clearance rate of the two groups [n (%)].

Bacterial name	Control-group				Observation-group			

	n	Completely clear	Partial clear	Clearance rate (%)	n	Completely clear	Partial clear	Clearance rate (%)
Escherichia coli	40	28	1	48.28%	42	37	2	62.71%
Klebsiella pneumoniae	8	4	2	6.90%	6	4	1	6.78%
Pseudomonas aeruginosa	5	3	1	5.17%	4	2	1	3.39%
Staphylococcus epidermidis	2	1	1	1.72%	3	2	1	3.39%
Staphylococcus aureus	1	1	0	1.72%	2	2	0	3.39%
Streptococcus	1	1	0	1.72%	1	1	0	1.69%
Others	1	0	1	0.00%	1	1	0	1.69%

Total	58	38	6	65.52%	59	49	5	83.05%

***Note:*** χ^2^=4.716, *P*<0.05.

**Table-IV:** Comparison of overall efficacy between the two groups [n (%)].

Group	n	Efficacy evaluation				Always effective

		cure	Markedly effective	efficient	invalid	
Control-group	55	12(21.82)	14(25.45)	18(32.73)	11(20.00)	44(80.00)
Observation-group	57	20(35.09)	22(38.60)	11(19.30)	4(7.01)	53(92.98)
*χ^2^*	-	-	-	-	-	4.067
*P*	-	-	-	-	-	0.044

## DISCUSSION

In this study, the effect of cefoperazone/sulbactam compound preparation in the treatment of patients with pyelonephritis was studied. Our results showed that cefoperazone/sulbactam at the ratio of 1:1 resulted in better clinical response, lower inflammatory reaction and higher bacterial clearance rate compared to 2:1 ratio, which was generally consistent with the findings of Chang et al.^9^

Pyelonephritis is mainly caused by bacterial infection, of which Escherichia coli is the most common, followed by *Proteus, Klebsiella and Enterobacter*.^11^ Cefoperazone sodium is a long-acting cephalosporin with a strong antibacterial effect on a variety of Gram-negative aerobic and anaerobic bacteria, but it has poor resistance to β-lactamases produced by various bacteria, such as Enterococcus and Pseudomonas aeruginosa.^15^

A study by Kuo et al.^16^ showed that sulbactam sodium can significantly enhance the antibacterial activity of cefoperazone against Serratia marcescens, Enterobacter cloacae and *Escherichia coli*. Wang L et al.^17^ also demonstrated that a stepwise increase in the ratio of sulbactam to partner β-lactam antibiotics led to a stepwise decrease in the minimum inhibitory concentrations (MICs) and a stepwise increase in the susceptibility rates. Ratio 1:3 of cefoperazone/sulbactam resulted in 91% sensitivity rate of *Acinetobacter Baumannii*. Lai CC et al.^18^ evaluated in-vitro activity of different cefoperazone sulbactam ratios on various multidrug resistant bacteria (MDROs), and showed that adding sulbactam enhanced cefoperazone activity against most MDROs excluding carbapenem-resistant P. Aeruginosa. The activity of cefoperazone-sulbactam against these MDROs was maximal at a ratio of 1:2, followed by ratios of 1:1 and 2:1.^18^ Therefore, clinically, for patients diagnosed with pyelonephritis, standard anti-infection treatment is often carried out first, and then the medication regimen are adjusted. In the actual clinical setting, although most patients can achieve a good curative effect, there are still cases of deterioration caused by a wrong selection of antibiotics.^19^

If pyelonephritis is not effectively controlled in time, it may lead to sepsis and septic shock, and even chronic renal failure. A study by Lee^20^ showed that the rate of septic shock in patients with bacteremic acute pyelonephritis was 26%, with a relatively high mortality rate. Elevated CRP levels are often seen in acute pyelonephritis and is considered a significant biomarker for the urinary infections.^21^,^22^ Mazaheri et al^12^ reported that serum levels of IL-6 and IL-8 are also sensitive biomarkers of urinary infections. Our study found that the levels of CRP, IL-6, IL-8 and WBC in the two groups decreased significantly after the treatment and was significantly lower in the observation group compared to the control group. Our results suggest that decreasing the proportion of cefoperazone is more efficient in improving the inflammatory responses of patients with pyelonephritis.

### Limitation of the study:

First, this was a retrospective study that relied on patients’ clinical records with limited data integrity. Second, the small sample size from only one single hospital makes the conclusions of the study less convincing.

## CONCLUSION

Clinical effect of 1:1 ratio of cefoperazone/sulbactam in the treatment of patients with pyelonephritis was better than that of 2:1. This ratio ensures full antibacterial effect of cefoperazone, with a significant decrease in inflammation markers and improved bacterial clearance.

### Authors’ contributions:

**WJ** conceived and designed the study.

**WJ** and **LC** collected the data and performed the analysis.

**WJ** was involved in the writing of the manuscript and is responsible for the integrity of the study.

All authors have read and approved the final manuscript.
